# Multi-Walled Carbon Nanotubes Impair Kv4.2/4.3 Channel Activities, Delay Membrane Repolarization and Induce Bradyarrhythmias in the Rat

**DOI:** 10.1371/journal.pone.0101545

**Published:** 2014-07-03

**Authors:** Xiao-Qiu Tan, Xiu-Li Cheng, Li Zhang, Bo-Wei Wu, Qing-Hua Liu, Jie Meng, Hai-Yan Xu, Ji-Min Cao

**Affiliations:** 1 Department of Physiology, Institute of Basic Medical Sciences Chinese Academy of Medical Sciences, School of Basic Medicine Peking Union Medical College, Beijing, China; 2 Department of Physiology, Shanxi Medical University, Taiyuan, China; 3 Department of Pathophysiology, Shanxi Medical University, Taiyuan, China; 4 Department of Biomedical Engineering, Institute of Basic Medical Sciences Chinese Academy of Medical Sciences, School of Basic Medicine Peking Union Medical College, Beijing, China; Temple University, United States of America

## Abstract

**Purpose:**

The potential hazardous effects of multi-walled carbon nanotubes (MWCNTs) on cardiac electrophysiology are seldom evaluated. This study aimed to investigate the impacts of MWCNTs on the Kv4/*I*_to_ channel, action potential and heart rhythm and the underlying mechanisms.

**Methods:**

HEK293 cells were engineered to express Kv4.2 or Kv4.3 with or without KChIP2 expression. A series of approaches were introduced to analyze the effects of MWCNTs on Kv4/*I*_to_ channel kinetics, current densities, expression and trafficking. Transmission electron microscopy was performed to observe the internalization of MWCNTs in HEK293 cells and rat cardiomyocytes. Current clamp was employed to record the action potentials of isolated rat cardiomyocytes. Surface ECG and epicardial monophasic action potentials were recorded to monitor heart rhythm in rats in vivo. Vagal nerve discharge monitoring and H&E staining were also performed.

**Results:**

Induction of MWCNTs into the cytosole through pipette solution soon accelerated the decay of *I*_Kv4_ in HEK293 cells expressing Kv4.2/4.3 and KChIP2, and promoted the recovery from inactivation when Kv4.2 or Kv4.3 was expressed alone. Longer exposure (6 h) to MWCNTs decreased the *I*_Kv4.2_ density, Kv4.2/Kv4.3 (but not KChIP2) expression and trafficking towards the plasma membrane in HEK293 cells. In acutely isolated rat ventricular myocytes, pipette MWCNTs also quickly accelerated the decay of *I*_Kv4_ and prolonged the action potential duration (APD). Intravenous infusion of MWCNTs (2 mg/rat) induced atrioventricular (AV) block and even cardiac asystole. No tachyarrhythmia was observed after MWCNTs administration. MWCNTs did not cause coronary clot but induced myocardial inflammation and increased vagus discharge.

**Conclusions:**

MWCNTs suppress Kv4/*I*_to_ channel activities likely at the intracellular side of plasma membrane, delay membrane repolarization and induce bradyarrhythmia. The delayed repolarization, increased vagus output and focal myocardial inflammation may partially underlie the occurrence of bradyarrhythmias induced by MWCNTs. The study warns that MWCNTs are hazardous to cardiac electrophysiology.

## Introduction

Carbon nanotubes (CNTs) have many potential applications in engineering science and medicine because of their controlled composition, electrical conductivity, and great tensile strength [1]. Meanwhile, scientists have been focusing on the biosecurity and toxicity of CNTs, a consideration which is necessary and meaningful for evaluating the reasonability and encouraging widespread application of CNTs in medicine [2]–[7]. One of the potential hazardous effects of CNTs is their impacts on ion channels and cardiac electrophysiology.

The toxicity of single-walled CNTs is greater than MWCNTs [8]. Therefore, MWCNTs may be more suitable in medical applications. We have reported previously that MWCNTs inhibited a variety of potassium currents including the transient outward current (*I*_to_), delayed rectifier K^+^ current (*I*_K_) and inwardly rectifying K^+^ current (*I*_K1_) in PC-12 cells [9]. This study focused on how MWCNTs affect the *I*_to_ channel and whether MWCNTs may exert an arrhythmogenic effect due to their repression on these K^+^ channels responsible for membrane repolarization.

*I*_to_ is an important channel current which contributes to the early-phase repolarization of action potentials especially in cardiomyocytes [10]. The triangular-shaped action potentials of rat cardiomyocytes suggest that a large *I*_to_ current is generated during the repolarizing phase in this type of cells. *I*_to_ consists of the fast transient outward current (*I*_to,f_) and the slow transient outward current (*I*_to,s_). *I*_to,f_ is the major component of *I*_to_ and is carried by a channel complex consisting of voltage-gated pore-forming subunits (Kv4.3 in humans, Kv4.2 and Kv4.3 in rodents) and an auxiliary subunit named potassium channel interacting protein 2 (KChIP2) [11], [12]. *I*_to_ modulates the action potential profile in cardiomyocytes, and regional differences in *I*_to_ contribute to the transmural ventricular electric heterogeneity. It has been confirmed that KChIP2 increases Kv4.2 current densities through enhancing the trafficking of Kv4 channel proteins from the endoplasmic reticulum to the surface membrane [13]. Some reports suggest that Kv4 and KChIP2 are downregulated in a variety of heart diseases [14]–[17]. Therefore, to evaluate the potential toxicity of CNTs on *I*_to_ channel, it is necessary to focus on the influences of CNTs on the expression and function of Kv4 and KChIP2. We also revealed previously that MWCNTs had no effect on mitochondrial membrane potential and Ca^2+^ mobilization in PC-12 cells [9], suggesting that MWCNTs suppress *I*_to_ currents likely through a direct disturbance on the Kv4-KChIP2 complex, other than by induction of oxidative stress. However, the exact mechanisms underlying the inhibitory effect of MWCNTs on *I*_to_ are unclear. In addition, inhibition of *I*_to_ channel (possibly also including other potassium channels [9]) may delay membrane repolarization and induce repolarization-associated cardiac arrhythmias, but these hypotheses need confirming. Using multiple techniques including electrophysiology, Western blotting, flow cytometry, co-inmmunoprecipitation and confocal microscopy, we addressed the effects of differentially modified MWCNTs (carboxylated (MWCNT-C), aminated (MWCNT-A) and pristine (MWCNT-P)) on the expression, trafficking and kinetics of Kv4.2/4.3 channels and the possible involvement of KChIP2 in engineered HEK293 cells. We also investigated the potential harms of MWCNTs on membrane repolarization and arrhythmogenesis in native cardiomyocytes and in hearts in vivo. The study may help to unveil the molecular mechanisms on how MWCNTs interfere with Kv4 channels and cardiac electrophysiology, and to pave the road for the biosecurity of CNTs application in nanomedicine.

## Materials and Methods

### Preparation and characterization of surface modified MWCNTs

We tested three types of MWCNTs in this study: the carboxylated MWCNTs (MWCNT-C), aminated MWCNTs (MWCNT-A) and the pristine (electrochemically neutral) MWCNTs (MWCNT-P). The prepared MWCNTs showed an outer diameter of 20–30 nm and a length range of 300 nm − 1.5 µm. The prepared MWCNTs were diluted in DMEM at a concentration of 20 µg/ml for cell study. For intravenous infusion, 1 mg/ml of MWCNTs were administrated to rats directly. For detailed information [18], see Text S1.

### Construction of plasmids

The expression plasmids pCMV-Script-mKv4.2 and pCMV-Script -mKChIP2 respectively containing genes of mouse Kv4.2 and KChIP2 were kind gifts from Prof. Rick Wilson (Washington University School of Medicine). pCMV6-Entry-hKv4.3 containing genes of human Kv4.3 tagged with Flag in the C-terminal was purchased from OriGene Technologies (USA). To generate the Flag-Kv4.2-GFP plasmid, a Flag tag (DYKDDDDK) was inserted into the extracellular loop between S1 and S2 of Kv4.2 using the overlapping PCR protocol and cloned in-frame into *Xho* I and *Eco*R I sites in the polylinker of pEGFP-N3 vector from Clontech (USA). Briefly, the first PCR fragment extended from the *Xho* I site to the Ser-212 codon in Kv4.2, plus a sequence encoding the DYKDDD portion of the Flag epitope (forward primer 1: 5′-CCGCTCGAGATGGCAGCCGGTGTTGCA-3′; reverse primer 1: 5′-TCGTCGTCCTTGTAGTCGCTAGACCCACATGG-3′). The second PCR fragment extended from the Ser-212 condon to the *Eco*R I site and contained the coding sequence for YKDDDDK (forward primer 2: 5′-CTACAAGGACGACGATGACAAGCCAGGCCACAT-3′; reverse primer 2: 5′-CCGGAATTCTCCAAGGCAGACACCCTGAC-3′) overlapping the first PCR fragment. A third round of PCR was performed using the acquired two fragments as templates and forward primer 1 and reverse primer 2 as primers. The resulting fragment was ultimately cloned into digested pEGFP-N3 vectors, producing the Flag-Kv4.2-GFP plasmid with Flag tag in the S1-S2 loop and GFP in the C-terminal of Kv4.2 protein. The plasmid was identified by DNA sequencing.

### Cell culture and transfection

Human embryonic kidney 293 (HEK293) cells were obtained from the Cell Center of Peking Union Medical College and cultured at 37°C in DMEM supplemented with 10% fetal bovine serum (FBS), 1 unit/ml penicillin, 100 mg/ml streptomycin and in an atmosphere of humidified 5% CO_2_ + 95% air. Cells were then transfected with 4 µg of expression plasmids using Lipofectamine 2000 (Invitrogen, USA) according to the manufacturer′s protocol. Stably expressing cell lines transfected with Flag-Kv4.2-GFP with or without pCMV-Script -mKChIP2 were selected and propagated for further study. The latter two cell lines were abbreviated as Kv4.2-KChIP2 and Kv4.2 cells, respectively.

### Isolation of cardiomyocytes

Rat left ventricular (LV) myocytes were routinely isolated by an enzymatic procedure [19]. Isolated cells were used for the recordings of *I*_to_ and action potentials. To isolate cardiomyocytes, male Sprague-Dawley rats (200–250 g) were anesthetized with an intraperitoneal injection of 10% chloral hydrate solution (0.3 ml/100 g). The heart was immediately harvested and plunged into ice-cold calcium-free Tyrode′s solution containing (in mM): NaCl 140, KCl 5.4, MgCl_2_ 1, NaH_2_PO_4_ 0.33, HEPES 10, and glucose 10. After mounting onto the Langendorff perfusion apparatus, the heart was perfused retrogradely through the aorta with calcium-free Tyrode′s solution and continuous oxygenation (95% O_2_ –5% CO_2_) for 10 min. Then, the perfusion was switched to a calcium-free, collagenase P (4–5 mg, Roche)-containing Tyrode′s solution (50 ml). After 10–20 min circular enzymatic digestion, the epicardial part of LV tissue was separated from the base of the heart, plated in KB solution and cut to small (about 1 mm^3^) blocks and stirred for cell isolation. The KB solution consisted of (in mM): KOH 85, L-glutamic acid 50, KCl 30, MgCl_2_ 1.0, KH_2_PO_4_ 30, glucose 10, taurine 20, HEPES 10, EGTA 0.5, pH 7.4 with KOH. Myocyte suspension was then filtered and cells were allowed to settle. The calcium concentration of the rinsing solution was gradually restored and finally to 1.8 mM. The isolated LV myocytes were used for patch clamp study.

### Western blotting

Western blotting was performed to determine the protein expression levels of Kv4.2/4.3 and KChIP2 in HEK293 cells at various conditions. HEK293 cells were scraped and collected by centrifugation at 1000 rpm for 5 min at 4°C and resuspended in a RIPA lysis buffer containing 50 mM Tris-Cl (pH 7.4), 150 mM NaCl, 1% Triton X-100, 1% sodium deoxycholate, 0.1% SDS and a series of protease inhibitor. After lysed thoroughly on ice, centrifugation was performed at 12000 rpm for 10 min at 4°C to acquire the supernatant. Protein quantity was determined by the BCA method and protein samples were aliquoted and stored at −80°C until use. Fifty micrograms of total proteins were separated by 10% SDS-PAGE and then transferred to PVDF membrane followed by blocking and incubation with anti-Kv4.2 (1∶1000, Abcam, UK), anti-Flag for Kv4.3 (1∶1000, Sigma, USA) or anti-KChIP2 (1∶2000, Abcam, UK) primary antibodies at 4°C overnight. Blots were then incubated with a horseradish peroxidase-conjugated secondary antibody (1∶5000) at room temperature for 1 h, and developed using the ECL system (Engreen Biosystem Co., Ltd., Beijing, China). Images were captured using the EC3 Imaging System (UVP, Inc., upland, CA, USA) and quantified by Quantity One software.

### Co-immunoprecipitation (co-IP)

Co-IP was conducted using Protein A/G PLUS Agarose Immunoprecipitation Reagent (Santa Cruz, USA). Briefly, (2−5)×10^7^ cells treated with different MWCNTs were lysed with mild lysis buffer (50 mM Tris-Cl, pH 7.4, 150 mM NaCl, 1 mM EDTA, 1% NP-40, 0.5% sodium deoxycholate) containing protease inhibitors. One microgram (µg) of primary antibody was applied and incubated at 4°C for 2 h. Then, the antibody-antigen complex was incubated overnight with protein A/G agarose at 4°C. The bead mixture was washed three times with cold PBS and finally resuspended with 2× SDS sample buffer, heated at 95°C for 5 min and centrifuged to acquire the supernatant. Samples were analyzed by Western blotting.

### Biotinylation assay and isolation of cell surface protein

Membrane protein biotinylation of transfected HEK293 cells was completed according to the protocol of Cell Surface Protein Isolation Kit (Pierce, USA). Briefly, transfected cells were incubated in PBS at 4°C to inhibit membrane protein internalization followed by incubation with 0.25 mg/ml Sulfo-NHS-SS-Biotin in PBS for 30 min on ice. After quenching the biotinylation reaction, cells were collected, lysed and purified by NeutrAvidin agarose. Finally, the labeled cell surface proteins which combined to beads were eluted using SDS-PAGE sample buffer and analyzed by Western blotting.

### Flow cytometry

Flow cytometry was used to investigate the trafficking of Kv4.2 to the membrane in HEK293 cells expressing Flag-Kv4.2-GFP with or without KChIP2 transfection according to Nesti's methods [20]. Since Flag tag was located at the extracellular S1-S2 loop, it could be used to detect the surface population of Kv4.2 by allophycocyanin (APC)-conjugated anti-Flag antibody, while GFP represented the total Kv4.2 level (including that at cytosole and membrane). To evaluate the effect of MWCNTs on Kv4.2 trafficking, cells were treated with MWCNTs (20 µg/ml) for 6 h, collected and washed with cold PBS three times. APC-conjugated anti-Flag antibody (1∶200, Abcam, UK) was applied and incubated in 4°C for 2 h followed by washing with cold PBS for three times. The fluorescence was detected using the flow cytometer (BD, USA) and the mean fluorescence intensity (MFI) was calculated with the following equation:
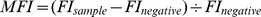



Here, *FI_sample_* is the fluorescence intensity of sample, *FI_negative_* stands for the fluorescence intensity of cells with no Flag-Kv4.2-GFP but incubated with APC conjugated anti-Flag antibody, *MFI_Flag_* is the expression level of Kv4.2 in membrane and *MFI_GFP_* indicates the expression level of Kv4.2 in whole cell. The ratio *MFI_Flag_/MFI_GFP_* is the percentage of membranous Kv4.2 over the total Kv4.2 protein, an indicator of Kv4.2 trafficking.

### Cell immunofluorescency

HEK293 cells expressing Flag-Kv4.2-GFP with or without KChIP2 were fixed by 4% paraformaldehyde for 15 min and blocked with 5% BSA for 1 h. For immunofluorescent staining of cytosolic Kv4.2 protein, 0.1% Triton-X 100 was used to permeabilize cell membrane. For staining membranous Kv4.2, Triton-X 100 was not used. After blocking with 5% BSA, cells were incubated with the primary mouse anti-Flag antibody (1∶1000, Sigma, USA) overnight at 4°C, washed, and then incubated with fluorescence 550-conjugated donkey anti-mouse secondary antibody (1∶200, Abcam, UK) for 1 h at room temperature. Immunofluorescence-labeled samples were examined under an Olympus confocal microscope with objectives of 60× oil immersion lens. The laser lines (excitation/emission wave) were 358 nm/461 nm, 488 nm/507 nm and 562 nm/576 nm for DAPI, GFP and fluorescence 550-conjugated mouse anti-Flag antibody, respectively. For negative control staining, the primary antibody was preincubated with the respective antigenic peptide (1∶1), this way cells did not show positive stain under the same staining procedures.

### Patch clamp

Voltage clamp mode was used to record Kv4.2 and Kv4.3 channel currents in HEK293 cells and transient outward current (*I*_to_) in acutely isolated ventricular myocytes. Current signals were acquired using the EPC-10 amplifier and PatchMaster software (Heka Elektronik, Lambrecht, Germany). For whole-cell recording, the junction potential between the intra- and extracellular solution was compensated by the EPC-10 amplifier. Series resistance (*R_s_*) was compensated by about 70% and the access resistance to <10 MΩ to minimize voltage errors. The experiments were conducted at room temperature (23±2°C). For recording of the Kv4.2/4.3 channel currents in HEK293 cells, the pipette solution consisted of (in mM): K-aspartate 100, KCl 40, NaCl 5, MgCl_2_ 5, HEPES 10, pH 7.2 with KOH. The bath solution consisted of (in mM): NaCl 140, KCl 5, MgCl_2_ 2, CaCl_2_ 2, HEPES 10, pH 7.4 with NaOH. For recording the *I*_to_ of rat ventricular myocytes, the pipette solution consisted of (in mM): K-aspartate 100, KCl 40, MgCl_2_ 1.0, HEPES 5.0, K_2_-ATP 3.0, pH 7.2 with KOH. The bath solution consisted of (in mM): NaCl 135, KCl 5.4, CaCl_2_ 1.8, MgCl_2_ 1.0, NaH_2_PO_4_ 0.33, HEPES 10, glucose 10, pH 7.3–7.4 with NaOH. CdCl_2_ (0.1 mM) and BaCl_2_ (0.2 mM) were added to the bath solution to block the *I*_Ca-L_ and *I*_K1_, respectively. The current and kinetics of Kv4 channel, including activation, inactivation, decay and recovery, were analyzed with corresponding protocols [21], [22]. A voltage step interval of 3 sec was used to insure channel recovery from inactivation. Specifically, to construct the activation curve, the conductance (*G*) was calculated first according to the equation 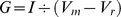, where *I* is the current amplitude at the testing membrane potential (*V_m_*) and *V_r_* is the reversal potential for potassium channels. The *G/G_max_-V_m_* curve, which reflects the voltage dependence of channel activation, was drawn according to the *G/G_max_* values at different *V_m_*. This curve was fitted by Boltzmann function and thus produced the half maximum activation potential (V_1/2-act_). To analyze the inactivation kinetics, we used *I/I_max_-V_m_* curve and fitted by Boltzmann function to acquire the half maximum inactivation potential (V_1/2-inact_). The decay time constants (τ_decay_) were acquired by fitting the traces recorded from −90 mV to +50 mV with single exponential function. The recovery curve was drawn according to *I/I_max_* values over different time durations. This curve was also fitted by exponential function getting the recovery time constant (τ_recovery_).

Current clamp mode was used to record the action potential of isolated rat ventricular myocytes in a whole-cell configuration. Short current pulses (800 pA, 1 ms) with a frequency of 1 Hz was delivered to the cell to induce action potentials. MWCNTs were added into the pipette solution to evaluate the effect of intracellular MWCNTs on action potentials, with the action potentials obtained under normal pipette solution as controls.

### Open-chest surgery and recordings of surface ECG and epicardial monophasic action potentials in rats in vivo

To observe the potential arrhythmogenic effect of MWCNTs in vivo, normal male Sprague-Dawley (SD) rats (200–250 g) underwent open-chest surgery in a sterile fashion. Briefly, animals were anesthetized with intraperitoneal injection of 10% chloral hydrate solution (0.3 ml/100 g), and air ventilated. Body temperature was maintained with a temperature-controlled operation table. Lead II surface electrocardiogram (sECG) was recorded throughout the experiment. Thoracotomy was performed and the heart was exposed. Monophasic action potentials (MAP) were recorded with a self-made unipolar recording electrode suggested by Irisawa [23] with some modifications. To make the recording electrode, a polyethylene tube (5 mm in diameter) was heated and soon drawn out by hands. The thin part of the tube was cut out with the stump diameter at 0.1−0.5 mm. Chloride-coated silver wire was inserted into the tube. The tube was then connected to a three-way stopcock which allowed for maintaining negative pressure after suction. Tyrode′s solution was introduced into the tube to immerse the silver wire so as to acquire the electrical signals without noise, and the reference electrode was connected to land. To observe the potential arrhythmogenic effect of MWCNTs, differently modified MWCNTs (2 mg/rat dispersed in 2 ml DMEM) was infused into the femoral vein within 2 min. Heart rhythm was continuously monitored by surface ECG and MAP.

### Recording of vagus discharge in vivo

This part of study was to observe the potential effect of MWCNTs on vagal tone. Detailed procedures were shown in Text S1.

### Transmission electron microscopy

Transmission electron microscopy (TEM) was performed to determine the internalization of MWCNTs in HEK293 cells and cardiomyocytes. Detailed procedures were shown in Text S1.

### Hematoxylin and eosin (H&E) staining

H&E staining was performed to examine the effect of MWCNTs on myocardial structures, including potential occurrence of coronary occlusion and inflammation. Detailed procedures were shown in Text S1.

The study including animal use protocol was approved by the Life Ethics Committee of Peking Union Medical College and in compliance with the U.S. National Institutes of Health Guidelines for the Care and Use of Laboratory Animals (NIH Publication 85–23).

### Statistical analysis

Numerical values were expressed as mean ± S.E.M. Student's *t* test for paired data and independent test were used for statistical analysis. Analysis of variance was used in case of multiple group comparisons. *P*<0.05 was considered statistically significant.

## Results

### The expressions of Kv4.2 and KChIP2 in HEK293 cells

**Figure 1:**
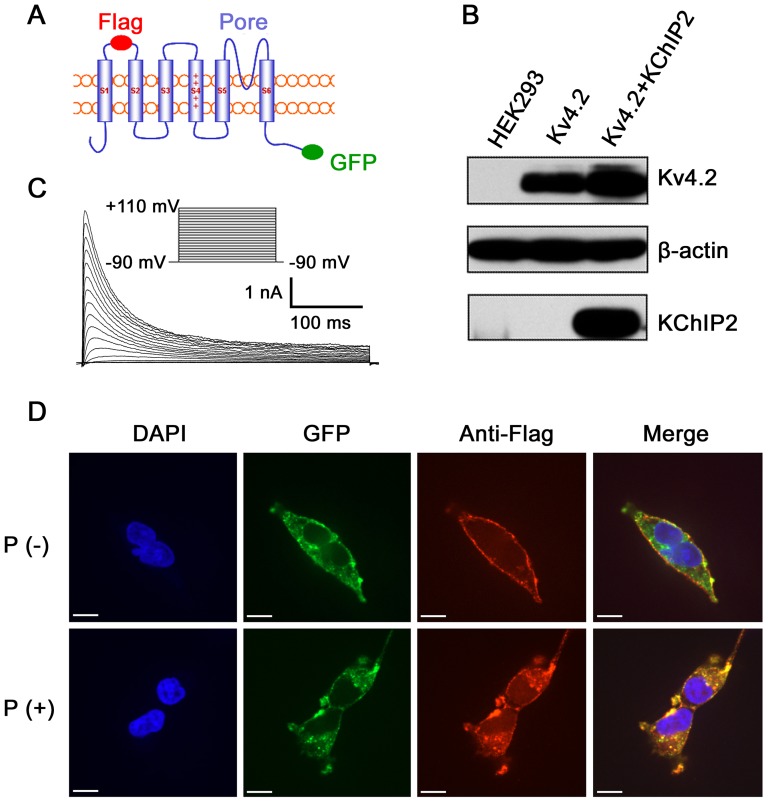
The expression of Kv4.2 and KChIP2 in HEK293 cells. ***A***, diagram for Flag-Kv4.2-GFP oriented in the plasma membrane. Flag and GFP were tagged in the extracellular S1-S2 loop and the C-terminal, respectively. ***B***, Western blotting analysis of HEK293 cells expressing Kv4.2 with or without the expression of KChIP2. The plasmids transfected in HEK293 cells were indicated above each lane. The detecting antibodies were indicated at the right side. Lane 1 showed the lysate from cells expressing the corresponding vector as control. Lane 2 and 3 represented cells expressing Kv4.2 alone or with KChIP2, respectively. ***C***, an example of *I*_Kv4.2_ recorded in HEK293 cells expressing Flag-Kv4.2-GFP. No significant difference of *I*_Kv4.2_ was found between Flag-Kv4.2-GFP-expressing cells and cells expressing untagged Kv4.2 (not shown). ***D***, confocal images of HEK293 cells transfected with Flag-Kv4.2-GFP, showing subcellular localization of Kv4.2, with (P (+)) or without (P (−)) membrane permeabilization. The surface population of Kv4.2 was visualized by anti-Flag antibody (*red*) and the whole Kv4.2 was indicated by GFP (*green*). The nuclei were stained by diamidino-phenyl-indole (DAPI) (*blue*). Note that anti-Flag antibody detected only the membranous Kv4.2 when without permeabilization, while it detected the overall Kv4.2 after permeabilization. The merged images showed perfect co-localization of Flag-Kv4.2-GFP detected by anti-Flag antibody and GFP, respectively. Scale bars  =  50 µm.

We constructed the Flag-Kv4.2-GFP plasmid and a successful expression of the Flag-Kv4.2-GFP protein was achieved in HEK293 cells. A diagram of Flag-Kv4.2-GFP oriented in the membrane was shown in Figure 1A. Flag and GFP were tagged in the S1-S2 extracellular loop and the C-terminal of Kv4.2, respectively. HEK293 cells expressing Kv4.2 alone or Kv4.2 plus KChIP2 were also indentified with Western blotting (Figure 1B). Importantly, we found that the presence of Flag and GFP tags did not affect the electrophysiological properties of Kv4.2 channel (Figure 1C and Table 1). Confocal microscopy showed that anti-Flag antibody detected only the surface population of Kv4.2 (Figure 1D). While after membrane permeabilization, anti-Flag antibody denoted the total Kv4.2 population, as indicated by the complete co-localization of Flag with GFP (Figure 1D). Thus, Flag tag was competent to examine surface expression of Kv4.2 when without membrane permeabilization, and Flag-Kv4.2-GFP is a valid and useful tool to study channel trafficking.

**Table 1:** Flag and GFP tags did not affect the kinetics of Kv4.2 channel in HEK293 cells.

cell line	n	V_1/2-act_ (mV)	V_1/2-inact_ (mV)	τ_decay_ (ms)	τ_recovery_ (ms)
Kv4.2	13	28.4±2.2	−25.5±0.4	41.5±2.1	160.4±17.2
Flag-Kv4.2-GFP	15	29.1±3.4	−27.4±0.5	43.1±2.5	153.9±9.3

The channel kinetics parameters of HEK293 cells expressing wild-type Kv4.2 or Flag-Kv4.2-GFP were examined in a whole-cell configuration. V_1/2-act_, membrane potentials at half maximum activation. V_1/2-inact_, membrane potentials at half maximum inactivation. τ_decay_, the decay time constant of inactivation. τ_recovery_, the recovery time constant from inactivation. These parameters between the two cell lines did not yield statistical significance.

### MWCNTs disturb the kinetics of Kv4.2/4.3 channels in HEK293 cells

**Figure 2:**
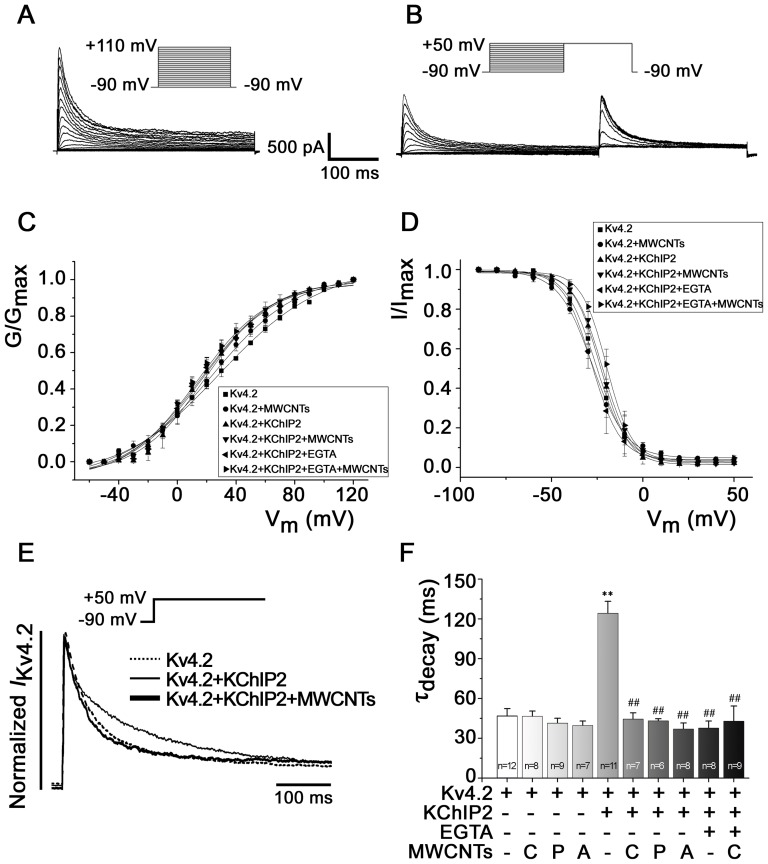
Effects of MWCNTs on the activation and inactivation kinetics of Kv4.2. ***A*** and ***B***, examples of activation (*A*) and inactivation (*B*) currents from transfected HEK293 cells. ***C*** and ***D***, Boltzmann equation-fitted activation and inactivation curves, respectively. ***E***, *I*_Kv4.2_ obtained by depolarizing the cell from −90 mV to +50 mV, showing accelerated decay upon MWCNTs treatment in HEK293 cells co-expressing Kv4.2 with KChIP2. ***F***, statistical data of decay time constants (τ_decay_) in various conditions. Abbreviations: C, carboxylated MWCNTs; P, pristine MWCNTs; A, aminated MWCNTs. ^**^ *P*<0.01 *vs.* Kv4.2. ^##^ *P*<0.01 *vs.* KChIP2.

We first tested the potential hazardous effect of MWCNTs (MWCNT-C, if not mentioned bellow) on the *I*_Kv4_ of HEK293 cells in a whole-cell configuration. Figures 2A and 2B show typical recordings of *I*_Kv4.2_ during activation and inactivation. Short time exposure (within 20 min) to MWCNTs (applied to bath solution) did not yield significant effect on the *I*_Kv4.2_ in HEK293 cells (data not shown). We then added MWCNTs to the pipette solution (intracellular application) to make a final concentration of 20 µg/ml. As shown in Figures 2C and 2D, pipette MWCNTs did not significantly affect the voltage dependence of Kv4.2 activation and inactivation under various conditions. However, pipette MWCNTs modulated the decay kinetics of Kv4.2 when KChIP2 was present (Figure 2E). KChIP2 slowed down the decay kinetics and intracellular calcium ion was necessary for this effect (Figure 2F), a phenomenon consistent with a previous report [24]. MWCNTs attenuated the effect of KChIP2 on the decay kinetics by shifting the decay time constant (τ_decay_) from 124.36±8.90 ms (n = 11) to 44.54±4.65 ms (n = 7) (*P*<0.01) (Figure 2F). However, pipette MWCNTs had no effect on the decay kinetics when KChIP2 was absent or intracellular free Ca^2+^ was chelated with 5 mM EGTA (Figure 2F). The three kinds of MWCNTs exerted similar effects on the decay kinetics of *I*_Kv4.2_ (Figure 2F).

**Figure 3:**
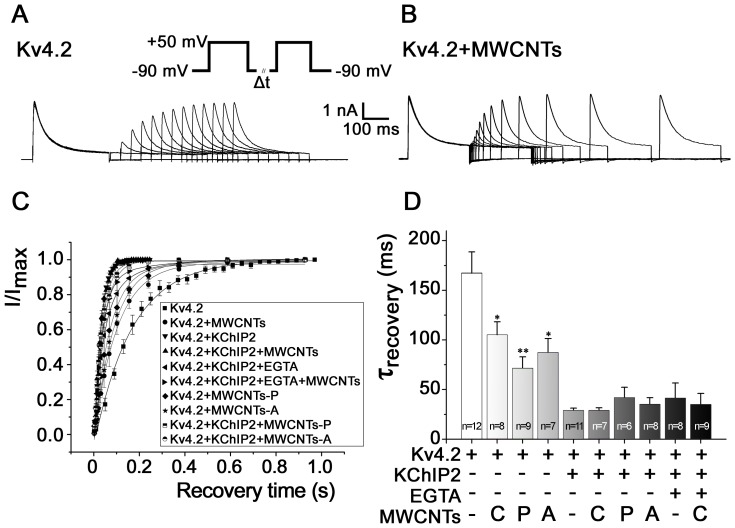
Effects of differently modified MWCNTs on the recovery kinetics of Kv4.2. MWCNTs were added to the pipette solution. ***A*** and ***B***, examples of recovery currents recorded from HEK293 cells expressing Kv4.2 with or without treatment of MWCNTs. ***C***, normalized *I*_Kv4.2_ in HEK293 cells upon different stimuli. ***D***, statistical data based on Figure 3C. * *P*<0.05, ** *P*<0.01 *vs.* Kv4.2.

We also analyzed the effect of MWCNTs on the recovery kinetics of Kv4.2 (Figure 3). Figures 3A and 3B show the original recovery *I*_Kv4.2_ currents recorded from HEK293 cells expressing Kv4.2 with or without treatment of MWCNTs. Pipette MWCNTs accelerated the recovery by shifting the recovery time constant (τ_recovery_) from 167.52 ± 21.16 ms (n = 12) to 105.24±13.05 ms (n = 8) (*P*<0.01) when Kv4.2 was expressed alone. However, MWCNTs had no remarkable effect on the τ_recovery_ when KChIP2 was co-expressed, with or without EGTA (Figures 3C and 3D), suggesting that intracellular Ca^2+^ is not necessary for KChIP2 to modulate the recovery kinetics of Kv4.2 channel. Pipette MWCNTs did not significantly affect the density of *I*_Kv4.2_ within 20 min (data not shown).

**Figure 4:**
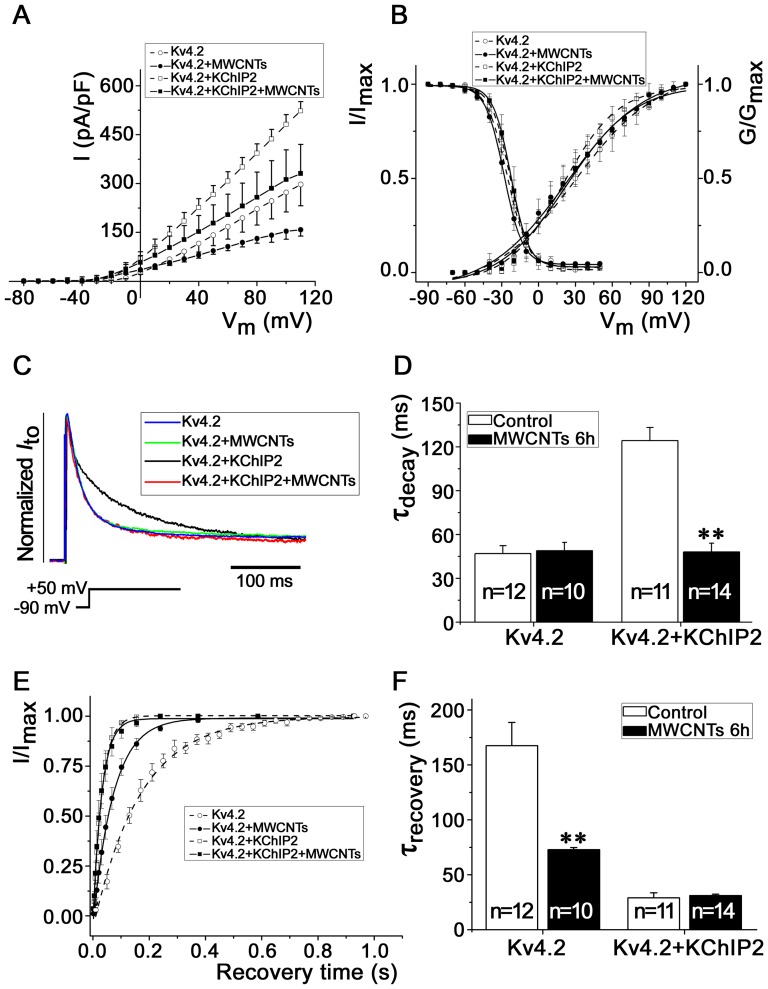
Effects of longer (6 h) exposure to MWCNTs on Kv4.2 channel kinetics in HEK293 cells. ***A***, I-V curves of *I*_Kv4.2_ at different conditions. ***B***, the voltage-dependent activation and inactivation curves fitted with Boltzmann equation. ***C*** and ***D***, effects of MWCNTs on the decay kinetics of HEK293 cells expressing Kv4.2 with or without KChIP2. ** *P*<0.01 *vs.* control. ***E*** and ***F***, the recovery curve and the statistical recovery time constant (τ_recovery_), respectively. ^**^ *P*<0.01 *vs.* control.

The above results showed that bath MWCNTs did not yield an acute effect on Kv4.2 channel kinetics but pipette MWCNTs did, these phenomena suggest that MWCNTs likely need to enter the cell to exert an influence on channel kinetics. With the help of TEM, we confirmed that MWCNTs could enter HEK293 cells and isolated rat cardiomyocytes after incubation for 6 h (Figure S1). Therefore, we applied MWCNTs in the culture medium for 6 h before the patch clamp experiments. Incubation with MWCNTs for 6 h reduced the current densities of *I*_Kv4.2_ from 523.07±28.77 pA/pF (n = 11) to 330.79±89.08 pA/pF (n = 14) (at V_m_ +110 mV), and from 297.46±65.38 pA/pF (n = 12) to 158.54±19.73 pA/pF (n = 10), in HEK293 cells expressing Kv4.2 with or without KChIP2, respectively (*P*<0.01) (Figure 4A). Similar with the effect obtained from pipette MWCNTs, incubation of HEK293 cells with MWCNTs for 6 h did not change the activation and inactivation curves of Kv4.2 (Figure 4B), but accelerated the decay kinetics, the τ_decay_ was shifted by MWCNTs from 124.36±8.90 ms (n = 11) to 48.01±6.10 ms (n = 14) (*P*<0.01) when Kv4.2 was co-expressed with KChIP2 (Figures 4C and 4D). Incubation with MWCNTs also accelerated the recovery kinetics by shifting the τ_recovery_ from 167.52±4.97 ms (n = 12) to 72.96±1.82 ms (n = 10) (*P*<0.01) in HEK293 cells expressing Kv4.2 alone (Figures 4E and 4F). These results indicate that longer exposure to MWCNTs could decrease the Kv4.2 current densities except for changing the channel kinetics, and further suggest that the *I*_Kv4.2_-suppressing effect of MWCNTs is likely associated with the decreased expression and trafficking of Kv4.2. This hypothesis is confirmed by the expression and trafficking experiments for Kv4.2 shown below.

We further compared the effects of the three kinds of modified MWCNTs (MWCNT-C, MWCNT-A and MWCNT-P) on Kv4.2 channel kinetics. The results showed that all of these MWCNTs had similar effects in accelerating the decay and recovery kinetics (Figures 2F, 3C and 3D), suggesting that the effects of MWCNTs on Kv4.2 kinetics are not due to the modification, but more likely related with their core structures and properties.

In addition, we investigated the effect of MWCNTs on the Kv4.3 channel expressed in HEK293 cells with or without KChIP2. The effects of MWCNTs on Kv4.3 channels were similar with that on Kv4.2 in shifting the channel kinetics (Figure S2). MWCNTs attenuated the effect of KChIP2 on the decay kinetics by shifting the τ_decay_ from 119.41±18.63 ms (n = 6) to 75.12±8.82 ms (n = 5) (*P*<0.01), but had no effect on the decay kinetics when KChIP2 was absent (Figures S2A and S2B). Besides, MWCNTs accelerated the recovery by shifting the τ_recovery_ from 175.91±26.20 ms (n = 7) to 112.69±19.07 ms (n = 6) (*P*<0.01) when Kv4.3 was expressed alone, but had no remarkable effect on the τ_recovery_ when KChIP2 was co-expressed (Figures S2C and S2D). As expected, MWCNTs did not significantly affect the voltage dependence of Kv4.3 activation and inactivation either expressed alone or with KChIP2 (data not shown).

### MWCNTs disturb the kinetics of *I*_to_ in isolated rat ventricular myocytes

**Figure 5:**
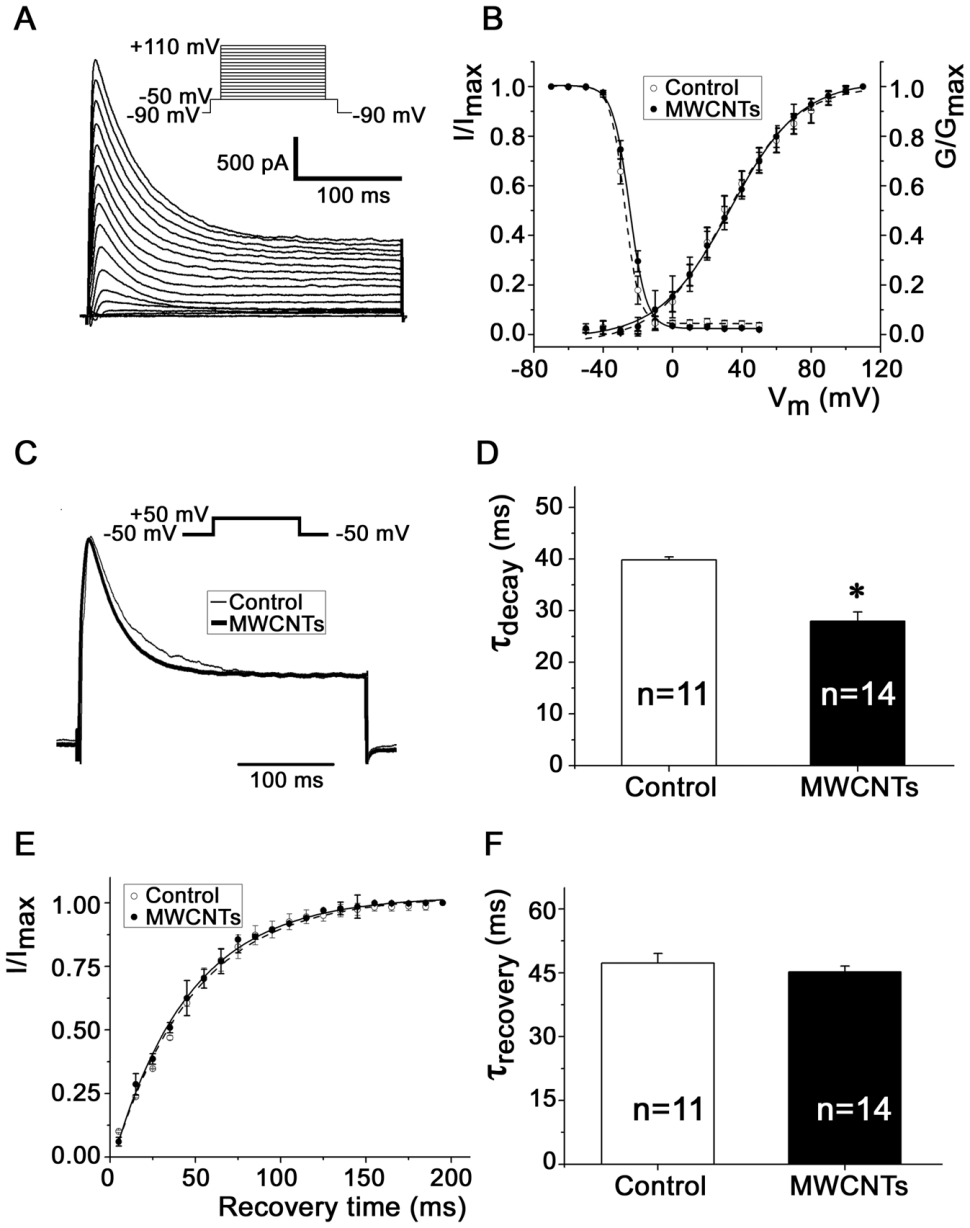
Effects of MWCNTs on the *I*_to_ of isolated rat ventricular myocytes. ***A***, a typical example of *I*_to_ recorded from ventricular myocytes. ***B***, voltage-dependent activation and inactivation curves of *I*_to_ from rat ventricular myocytes with or without (control) MWCNTs (20 µg/ml) incubation. ***C***, comparison of the *I*_to_ traces and τ_decay_ in rat ventricular myocytes with (*thick*) or without (*thin*) MWCNTs treatment. Ventricular myocytes were depolarized from −50 mV to +50 mV. ***D***, statistical data of the τ_decay_ of ventricular myocytes with or without MWCNTs treatment. ***E***, *I*_to_ recovery curves. ***F***, statistical τ_recovery_ bar graphs of control cells and MWCNT-treated cells. * *P*<0.05 *vs.* control.

As *I*_Kv4.2_ is the major component of *I*_to_ in rat ventricular myocytes, we determined whether the effect of MWCNTs on *I*_Kv4.2_ in HEK293 cells would also recur in the native ventricular myocytes. Figure 5A shows the typical *I*_to_ recorded from native ventricular myocytes. Similar with that observed in HEK293 cells, MWCNTs did not affect the voltage dependence of activation and inactivation kinetics of ventricular myocytes (Figure 5B). MWCNTs accelerated the decay kinetics of inactivation of rat ventricular myocytes, with a shift of τ_decay_ from 39.81±0.58 ms (n = 11) to 27.97±1.77 ms (n = 14) (*P*<0.05) (Figures 5C and 5D). However, MWCNTs did not yield a significant effect on the recovery kinetics of *I*_to_ channels in cardiomyocytes which naturally express Kv4.2 and KChIP2 (Figure 5E). This phenomenon is consistent with that in HEK293 cells co-expressed with Kv4.2 and KChIP2 (shown in Figures 4E and 4F).

### MWCNTs suppress the expressions of Kv4.2/4.3 but not of KChIP2 in HEK293 cells

**Figure 6:**
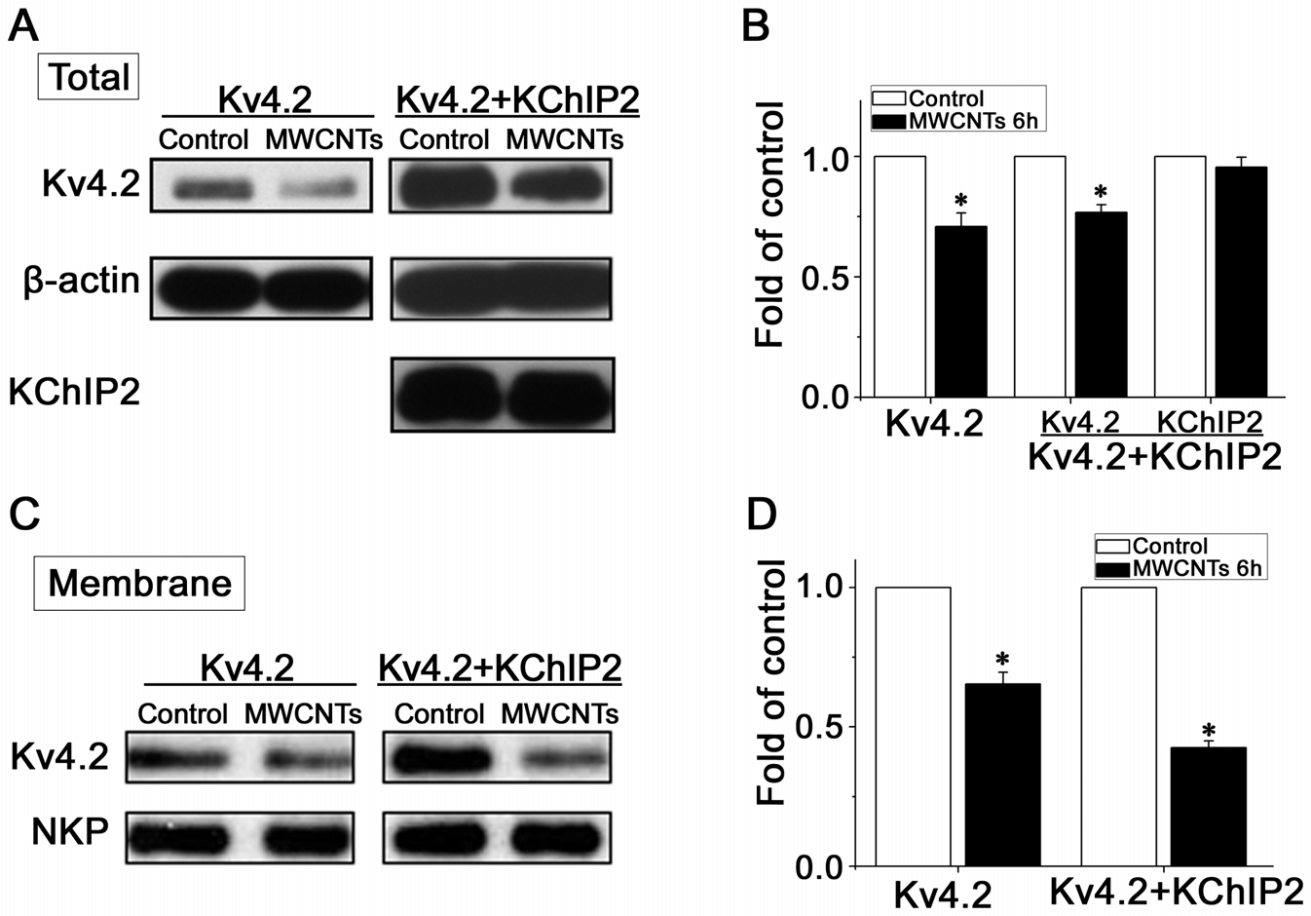
Effects of MWCNTs on Kv4.2 and KChIP2 protein expression in transfected HEK293 cells. ***A***, Western blotting analysis showing changes of Kv4.2 and KChIP2 in two cell lines. ***B***, statistical histograms from Figure 6A showing the fold changes after 6 h-treatment with MWCNTs. ***C***, biotinylation results showing the surface population of Kv4.2, with the expression of endogenous Na^+^/K^+^-ATPase (NKP) as loading control. ***D***, quantification histograms indicating the fold changes of the surface population of Kv4.2. * *P*<0.05 *vs*. control.

There is a possibility that MWCNTs affect not only Kv4 channel kinetics, but also channel protein expression. To address this hypothesis, we checked the effects of MWCNTs on the protein expressions of Kv4.2/4.3 and KChIP2 using Western blotting (Figures 6 and S3). Incubation of HEK293 cells with MWCNTs (20 µg/ml) for 6 h decreased the expression of Kv4.2 in HEK293 cells, no matter KChIP2 was co-expressed with Kv4.2 or not (Figure 6). However, MWCNTs did not affect the KChIP2 expression level (Figures 6A and 6B). In addition, we detected the effect of MWCNTs on the membranous expression level of Kv4.2 with the biotinylation assay. The results showed that MWCNTs significantly reduced the surface Kv4.2 level of HEK293 cells with or without KChIP2 co-expression (Figures 6C and 6D), indicating that MWCNTs suppressed the expression of Kv4.2 not through decreasing the expression of KChIP2 at least in engineered HEK293 cells. We also found that MWCNTs decreased the protein expression level of Kv4.3 expressed in HEK293 cells without affecting the expression of KChIP2 (Figures S3A and S3C). This was also consistent with that on Kv4.2.

### MWCNTs inhibit Kv4.2 trafficking towards cell membrane via KChIP2 in HEK293 cells

**Figure 7:**
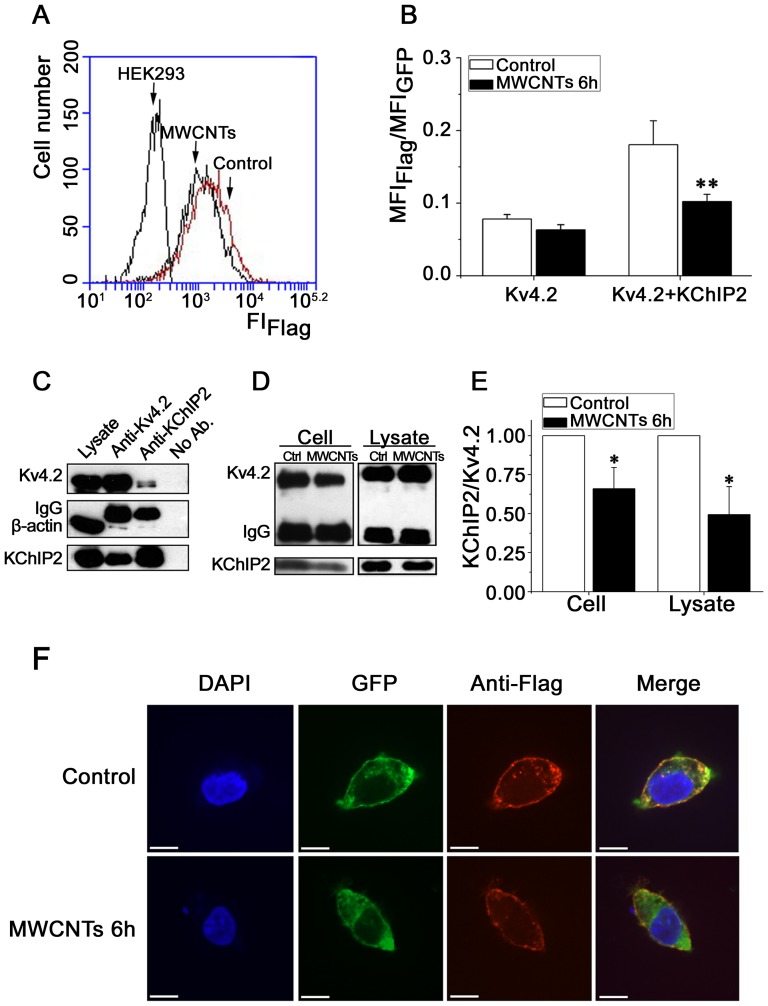
Effects of MWCNTs on Kv4.2 trafficking in HEK293 cells. ***A***, an original recording of flow cytometry showing the fluorescence of Flag which represented the population of Kv4.2 in cell membrane. A leftward shift of the trace meant a reduction of fluorescence intensity in membrane and vise versa. Untransfected HEK293 cells served as negative control. ***B***, ratio of mean fluorescence intensity of Flag to GFP in HEK293 cells expressing Kv4.2 with or without KChIP2 upon MWCNTs treatment from Figure 7A. ***C***, aliquots of cell lysate from HEK293 cells expressing Kv4.2 and KChIP2 were mixed with either anti-Kv4.2 antibody or anti-KChIP2 antibody and precipitated with protein A-sepharose. Immune complexes were resolved by SDS-PAGE. The immunoprecipitating antibodies were indicated above each lane, and detecting antibodies were shown at the left side. ***D***, effects of MWCNTs treatment on the ratio of KChIP2 to Kv4.2 with anti-Kv4.2 antibody as bait for co-IP in HEK293 cells and cell lysate. MWCNTs were applied to the culture medium and cell lysate for 6 h, respectively. ***E***, statistical results of the effects of MWCNTs on the ratio of KChIP2 to Kv4.2 in HEK293 cells expressing Kv4.2 and KChIP2. * *P*<0.05, ** *P*<0.01 *vs.* control. ***F***, confocal images showing the membranous and intracellular distributions of Kv4.2. GFP (*green*) and anti-Flag *(red*) represented the total and membranous Kv4.2, respectively. Note that MWCNT-treated cells showed reduced membranous Kv4.2 compared with control cells. Scale bars  =  50 µm.

Based on above results that MWCNTs interfered with Kv4.2 channel kinetics, current densities and membranous expression level, we hypothesized that MWCNTs may also damage Kv4.2 trafficking towards the cell membrane. We used several experimental assays, including flow cytometry, co-immunoprecipitation and confocal microscopy, to address this hypothesis. From the results of flow cytometry (Figures 7A and 7B), MWCNTs (20 µg/ml) decreased the ratio of membranous Kv4.2 (Flag) and total Kv4.2 (GFP) from 0.18±0.03 to 0.10±0.01 (n = 4, *P*<0.01) in HEK293 cells co-expressed with Kv4.2 and KChIP2. However, when Kv4.2 was expressed alone, this ratio did not show significant difference between MWCNT-treated (0.06±0.01) and untreated (0.08±0.01) HEK293 cells (*P*>0.05) (Figure 7B). Using the co-IP assay, we investigated the protein-protein interaction of Kv4.2 and KChIP2 upon MWCNTs treatment (Figures 7C, 7D and 7E). As MWCNTs reduced the expression of Kv4.2 without affecting the expression of KChIP2 (Figure 6A), we added MWCNTs in the culture medium and incubated for 6 h, or added MWCNTs to the cell lysate for 6 h. The level of Kv4.2 was used to normalize and calculate the content of KChIP2 co-immunoprecipitated by anti-Kv4.2 antibody, an indicator denoting the ability of Kv4.2 interacting with KChIP2. Figures 7D and 7E showed that MWCNTs (20 µg/ml) reduced the level of anti-Kv4.2-immunoprecipitated KChIP2, the ratio of KChIP2/Kv4.2 was reduced by MWCNTs to (66.0±13.6)% (*cell*) (n = 4, *P*<0.05), and to (49.4±18.0)% (*lysate*) (n = 4, *P*<0.05), respectively. MWCNTs exerted similar inhibitory effect on the interaction between Kv4.3 and KChIP2 (Figures S3B and S3D). These results indicate that MWCNTs inhibited the interactions between Kv4.2/4.3 and KChIP2. In the confocal microscopy study (Figure 7F), GFP (*green*) and anti-Flag (*red*) represented the total and membranous Kv4.2, respectively. MWCNTs (20 µg/ml) exposure for 6 h reduced the membranous Kv4.2 compared with the control cell (Figure 7F, *red*). Taken together, these data proved our hypothesis that MWCNTs damage Kv4.2 trafficking towards cell membrane via inhibiting the interaction between Kv4.2 and KChIP2.

### MWCNTs prolong the action potential duration of isolated ventricular myocytes and induce bradyarrhythmias in rats in vivo

**Figure 8:**
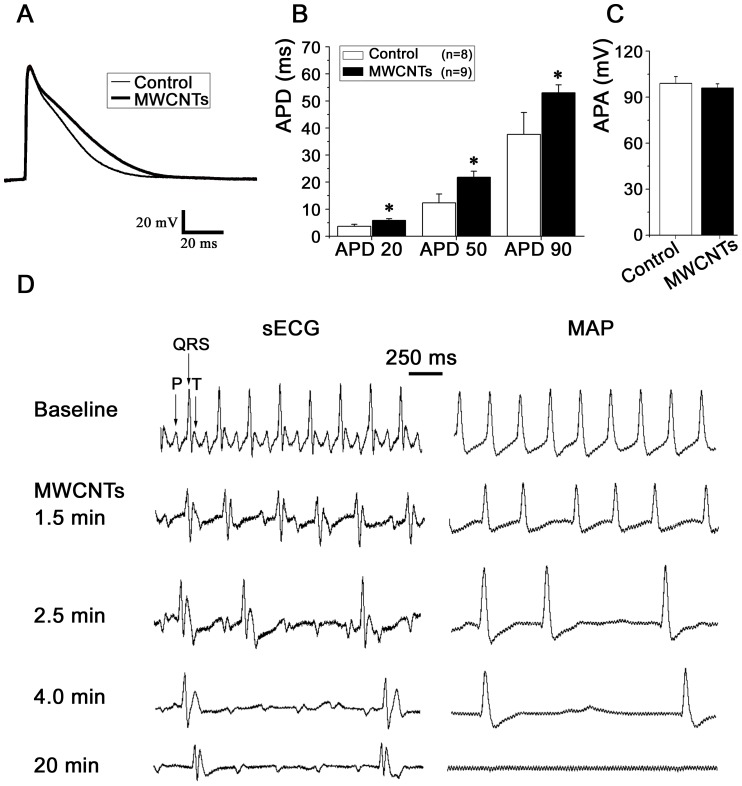
The action potential-shaping and arrhythmogenic effects of MWCNTs. ***A***, an overlap of the action potentials of isolated ventricular myocytes at baseline and after MWCNT treatment. ***B*** *and* ***C***, statistical data showing the effects of MWCNTs on the APD and APA, respectively. ***D***, typical surface ECGs and ventricular MAPs recorded from an in-situ heart showing that intravenous MWCNTs soon induced bradycardia, AV block and cardiac asystole. P, QRS and T represented the P wave, QRS complex and T wave, respectively.

As *I*_to_ contributes to the early repolarization of cardiomyocyte action potential and MWCNTs suppress *I*_to_ and some other potassium channels, we speculated that MWCNTs would prolong the action potential duration (APD) of native cardiomyocytes. As expected, MWCNTs of 20 µg/ml applied into pipette solution prolonged the APD of isolated rat ventricular myocytes (Figures 8A and 8B) compared with the control cells. APD20 was prolonged by MWCNTs from 3.64±0.78 ms (n = 8) to 5.84±0.62 ms (n = 9), APD50 from 12.35±3.23 ms to 21.81±2.22 ms and APD90 from 37.65±8.07 ms to 53.03±2.94 ms (all *P*<0.05). MWCNTs did not significantly affect the action potential amplitude (APA) (control cell 95.89±2.78 mV *vs.* MWCNT-treated cell 98.98±4.39 mV, *P*>0.05) (Figure 8C).

Intravenous infusion of MWCNTs (2 mg/rat dispersed in 2 ml DMEM) induced bradyarrhythmias, the heart rate began to slow down and showed subsequent sinus arrhythmia 1.5 min after the infusion (Figure 8D), then sequentially developed to bradycardia, atrioventricular (AV) block and in some rats, regional ventricular conduction block or cardiac arrest (Figure 8D), as shown that local MAP could not be detected (Figure 8D) and the heart beat became very weak or even stopped beating, though surface ECG still reflected AV block about 20 min after MWCNTs infusion (Figure 8D). These phenomena suggest cardiac conduction block, electro-mechanical dissociation and cardiac asystole. No significant difference was found among the three kinds of MWCNTs in their ability to induce bradyarrhythmias. A total of 18 rats were infused with MWCNTs, most (15/18) of them showed heart rate slowing, 10 of the 18 rats showed AV block and 5/18 developed cardiac asystole after MWCNTs infusion. Control rats (placebo DMEM (2 ml i.v.) without MWCNTs) showed normal heart rhythm throughout the experiments (data not shown). Tachyarrhythmia was not observed after infusion of MWCNTs in normal rats.

We further investigated the potential mechanisms by which MWCNTs induced bradyarrhythmias at the integrative level. MWCNTs increased vagus discharge about 3–4 min after administration (Figures S4A and S4B). In addition, the H&E stains showed that MWCNTs did not induce coronary clot (Figures S4C and S4D) but induced inflammatory cell infiltration in the myocardium shortly (about 10 min) after MWCNTs administration (Figures S4E and S4F). Taken together, the increased vagal output, myocardial inflammation and APD prolongation may at least partially account for the MWCNT-induced bradyarrhythmias.

## Discussion

The present study focused on the impacts and mechanisms of MWCNTs on *I*_to_ channel, action potential and arrhythmogenesis. We first investigated the effects of MWCNTs on the kinetics, current density, expression and trafficking of Kv4.2/4.3 channel in HEK293 cell line with or without KChIP2 expression. We further observed the influences of MWCNTs on the *I*_to_ channel and action potential of rat ventricular myocytes and arrhythmogenesis in rats in vivo. The results revealed that MWCNTs did exert some influences on Kv4/*I*_to_ channel current, kinetics, trafficking and expression, and prolonged the APD of cardiomyocytes and induced bradyarrhythmias in vivo. These results suggest that MWCNTs could indeed impair the activities of Kv4/*I*_to_ channels and the stability of cardiac electrophysiology. The study demonstrated for the first time that MWCNTs are “toxic” to cardiac electrophysiology, just as CNTs being classified “toxic” in terms of nanoecotoxicology [25].

The patch clamp data showed that MWCNTs likely need to enter the cell to exert an interfering effect on Kv4 channel kinetics, and implying that the action site of MWCNTs likely locates at the intracellular side of the plasma membrane. The TEM study confirmed that MWCNTs could enter both the HEK293 cells and cardiomyocytes within 6 h. The TEM images further indicate that MWCNTs could directly “insert” into the cytoplasm of cardiomyocytes instead of endocytosis which was characterized by the endosome. While the internalization of MWCNTs in HEK293 cells was mostly accomplished by endocytosis, as MWCNTs usually presented inside vesicles, with some MWCNTs located barely in the cytoplasm.

KChIPs are calcium-binding proteins with four EF-hands Ca^2+^-binding motifs, and belong to the neuronal calcium-sensor (NCS) superfamily [26], [27]. KChIP2 is the predominant isoform of KChIPs in the heart [28]. It has been confirmed that KChIP2 possesses two main effects in modulating the inactivation and recovery kinetics and increasing the expression and trafficking of Kv4 channels to the plasma membrane through interaction with Kv4 [29], [30]. Structure analysis showed that the α-helix of NH_2_-terminal motifs (amino acid residues 1–30) of Kv4.2 was closely contacted with KChIP1 within a hydrophobic groove, and formed a stable complex [31], [32]. Distinct regions of KChIP were involved in modulating the inactivation and recovery of Kv4.3 [24]. Here, MWCNTs accelerated the decay kinetics of Kv4/*I*_to_ channels both in HEK293 cells and cardiomyocyte, suggesting an interference of MWCNTs on KChIP2 or Kv4-KChIP2 interactions.

One major function of KChIP2 is to slow down the inactivation kinetics and accelerate the recovery kinetics of Kv4 channel [11]. We showed here that KChIP2 slowed the inactivation of Kv4.2/4.3 channels in HEK293 cells and accelerated the recovery from inactivation compared with cells expressing Kv4.2 or Kv4.3 alone. It is known that intracellular calcium ion is necessary for KChIP2 to modulate the inactivation kinetics of Kv4 channels, but is unnecessary for modulating the recovery kinetics [24]. In the present study, an intracellular calcium-free condition was created by adding 5 mM EGTA in the pipette solution. Under this condition, the decay time constant (τ_decay_) of inactivation of Kv4.2 channel was shortened by MWCNTs from about 124 ms to 38 ms in cells co-expressed with Kv4.2 and KChIP2, a τ_decay_ similar with that from HEK293 cells expressed Kv4.2 alone (about 47 ms). We also showed that MWCNTs accelerated the decay kinetics when Kv4.2 was co-expressed with KChIP2. However, MWCNTs could not accelerate the decay when Kv4.2 was expressed alone or intracellular EGTA (5 mM) was applied, suggesting that MWCNTs attenuated the effect of KChIP2 on Kv4.2 channel inactivation. Additionally, MWCNTs accelerated the recovery from inactivation when Kv4.2 was expressed alone, while did not affect the recovery kinetics when KChIP2 was co-expressed. This might be due to a direct interaction of MWCNTs with Kv4.2, and the interacting site may be different from that for KChIP2 to modulate the inactivation. We also showed that differently modified MWCNTs (carboxylated, aminated and pristine) exerted similar effects on the inactivation and recovery kinetics of Kv4.2, suggesting that these modulations do not significantly contribute to the effect of MWCNTs on Kv4.2 channel kinetics.

Except for KChIP2, some other auxiliary subunits, such as DPPX, minK and Kvβ, may also modulate the function of Kv4 in native cells. We showed that the *I*_to_ inactivation kinetics of native cardiomyocytes was much faster than that of HEK293 cells co-expressed with Kv4.2 and KChIP2. One reason may be that these additional auxiliary subunits accelerate Kv4.2 inactivation in native cells, as reported that DPPX accelerates the inactivation of Kv4.2 [33]. MWCNTs expedited the inactivation kinetics in ventricular myocytes as in HEK293 cells which do not express these auxiliary subunits, suggesting that the effect of MWCNTs on Kv4.2 inactivation kinetics is due to a disturbance of MWCNTs on the interaction between Kv4.2 and KChIP2. The similarities and differences between the channel kinetics-modulating effects of MWCNTs in HEK293 cells and native cardiomyocytes may be owing to the heterogeneities of the subcellular machinenaries including Kv4.2 auxiliary subunits. Nevertheless, cell line studies helped us to explore the action mechanisms of MWCNTs on Kv4.2 channel and action potential.

A second known effect of KChIP2 on Kv4 is to increase the expression and trafficking of Kv4 [13]. This effect of KChIP2 was reconfirmed in this study. We first observed that MWCNTs suppressed the expressions of Kv4.2/4.3 channels. Interestingly, MWCNTs did not affect the expression of KChIP2 protein. In addition, MWCNTs inhibited the trafficking of Kv4.2 in HEK293 cells. As KChIP2 interacts with Kv4 via the combination of its C-terminal with the N-terminal of Kv4 [24], we used co-IP to detect the effect of MWCNTs on the interaction between Kv4.2/Kv4.3 and KChIP2, and showed that MWCNTs decreased the binding level of KChIP2 with Kv4.2/Kv4.3. After the treatment of both cells and cell lysate with MWCNTs for 6 h, the ratio of KChIP2 binding to Kv4.2 was decreased compared with the control. This result suggests that MWCNTs attenuated the interaction between Kv4.2/Kv4.3 and KChIP2, this was consistent with the result that MWCNTs accelerated the inactivation kinetics. These results suggested that the reduction of current density after longer MWCNTs exposure is likely due to suppressions of Kv4 expression and trafficking by MWCNTs. As endocytosis is an important mechanism in regulating the expression level of channels in the plasma membrane, another possibility is that MWCNTs incubation may increase the rate of endocytosis and thus decrease the current densities.

Based on the present data, we feel that the action potential-shaping effect of MWCNTs at the cellular level and the arrhythmogenic effect of MWCNTs at the integrative level can not be totally owing to the effect of MWCNTs on Kv4.2/*I*_to_ channel. First, MWCNTs may affect several potassium channels [9] and even other cell machineries, not just *I*_to_ channel. Suppression of *I*_K_ and *I*_K1_ channels by MWCNTs [9] may also rationally contribute to APD prolongation in cardiomyocytes if confirmed. Second, we showed that MWCNTs need to enter the cell to affect the *I*_to_ channel, while the endocytotic process for nanomaterials entering cells with low phagocytotic capacity usually takes an hour or longer [34]. However, MWCNTs intravenous infusion soon (within minutes) induced bradyarrhythmias. These phenomena suggest that MWCNTs may exert more complicated effects at the integrative level than its cellular effects in vitro.

To further investigate the potential mechanisms by which MWCNTs induce bradyarrhythmias, we performed a series of experiments at both the cellular level and integrative level. At the cellular level, MWCNTs induced APD prolongation which favors the occurrence of AV block and other types of bradyarrhythmias. At the integrative level, MWCNTs increased vagus output and myocardial inflammation within a short time span. We considered that APD prolongation, increased vagus output and myocardial inflammation may at least in part underlie the MWCNT-induced bradyarrhythmias. While potential coronary clot is not a cause, as the H&E stain did not show coronary occlusion and the ECG did not imply any sign of cardiac ischemia.

Conclusions and clinical implications. MWCNTs impair the Kv4.2/4.3 channel activities with complicated mechanisms including a direct action on Kv4 channels and an interfering on Kv4 channel and KChIP2 interaction. These cellular/molecular effects of MWCNTs, plus their effects at the integrative level, may underlie the pathogenesis of bradyarrhythmias after MWCNTs administration. The study raises a warning in situations of CNT exposures, such as medical practice using MWCNTs as a drug delivery tool, air and water pollution with CNTs in special sites and accidental intake of CNTs, that CNTs are toxic to animals and potentially to humans, especially on the heart.

## Supporting Information

Figure S1 **Transmission electron microscopy (TEM) images showing the internalization of MWCNTs in HEK293 cells and cardiomyocytes.** ***A***, extracellular MWCNTs showing the appearance of this type of CNT under the transmission electron microscope (arrows). ***B*** and ***C***, MWCNTs presented inside the endosomes of HEK293 cells (arrows). ***D***, MWCNTs presented in the cytoplasm of HEK293 cells (arrows). ***E*** and ***F***, MWCNTs located in the cytoplasm of rat LV cardiomyocytes (arrow heads). Scale bars, 200 nm.
(TIF)

Figure S2 **Effects of MWCNTs on the electrophysiological properties of Kv4.3 channel.** ***A***, an overlap of normalized *I*_Kv4.3_ showing the effects of MWCNTs on the decay kinetics in HEK293 cells expressing Kv4.3 alone or with KChIP2. ***B***, statistical data of decay time constants (τ_decay_) based on Figure S2A. ***C***, recovery curve fitted by exponential function showing the effects of MWCNTs on the recovery kinetics in HEK293 cells expressing Kv4.3 alone or with KChIP2. ***D***, the statistical recovery time constant (τ_recovery_) based on Figure S2C. * *P*<0.05 *vs*. control.
(TIF)

Figure S3 **Effects of MWCNTs on the expression of Kv4.3 and on the interaction between Kv4.3 and KChIP2.** ***A*** and ***C***, Western blotting analysis showing changes of Kv4.3 and KChIP2 in two cell lines and statistical histograms from Figure S3A showing the fold changes after 6 h-treatment with MWCNTs. ***B***, co-IP assay showing the effect of MWCNTs on the interaction between Kv4.3 and KChIP2. The immunoprecipitating antibody (anti-Flag for Kv4.3) was indicated above the lane, and detecting antibodies were shown at the left side. ***D***, statistical results of the effects of MWCNTs on the ratio of KChIP2 to Kv4.3 in HEK293 cells expressing Kv4.3 and KChIP2. ** *P*<0.01 *vs.* control. n = 4 for each experiment.
(TIF)

Figure S4 **Recording of right cervical vagus discharges and the H&E staining images of rat LV tissues before and after MWCNTs administration in vivo.** ***A*** and ***B***, vagal discharges before and 3 min after MWCNTs administration in rats in vivo, respectively. Note that vagal discharge was increased by MWCNTs. ***C*** through ***F***, hematoxylin and eosin (H&E) staining of rat LV myocardium showing that MWCNTs did not induce coronary occlusion but induced focal myocardial inflammation 10 min after MWCNTs administration.
(TIF)

Text S1 Supplementary materials and methods (see in a separate file).
(DOC)
