# Supplementary figures and images for: Multi-Walled Carbon Nanotubes Impair Kv4.2/4.3 Channel Activities, Delay Membrane Repolarization and Induce Bradyarrhythmias in the Rat
Source: PLoS One. 2014 Jul 3;9(7):e101545. doi: 10.1371/journal.pone.0101545 (PMC4081717; doi:10.1371/journal.pone.0101545)

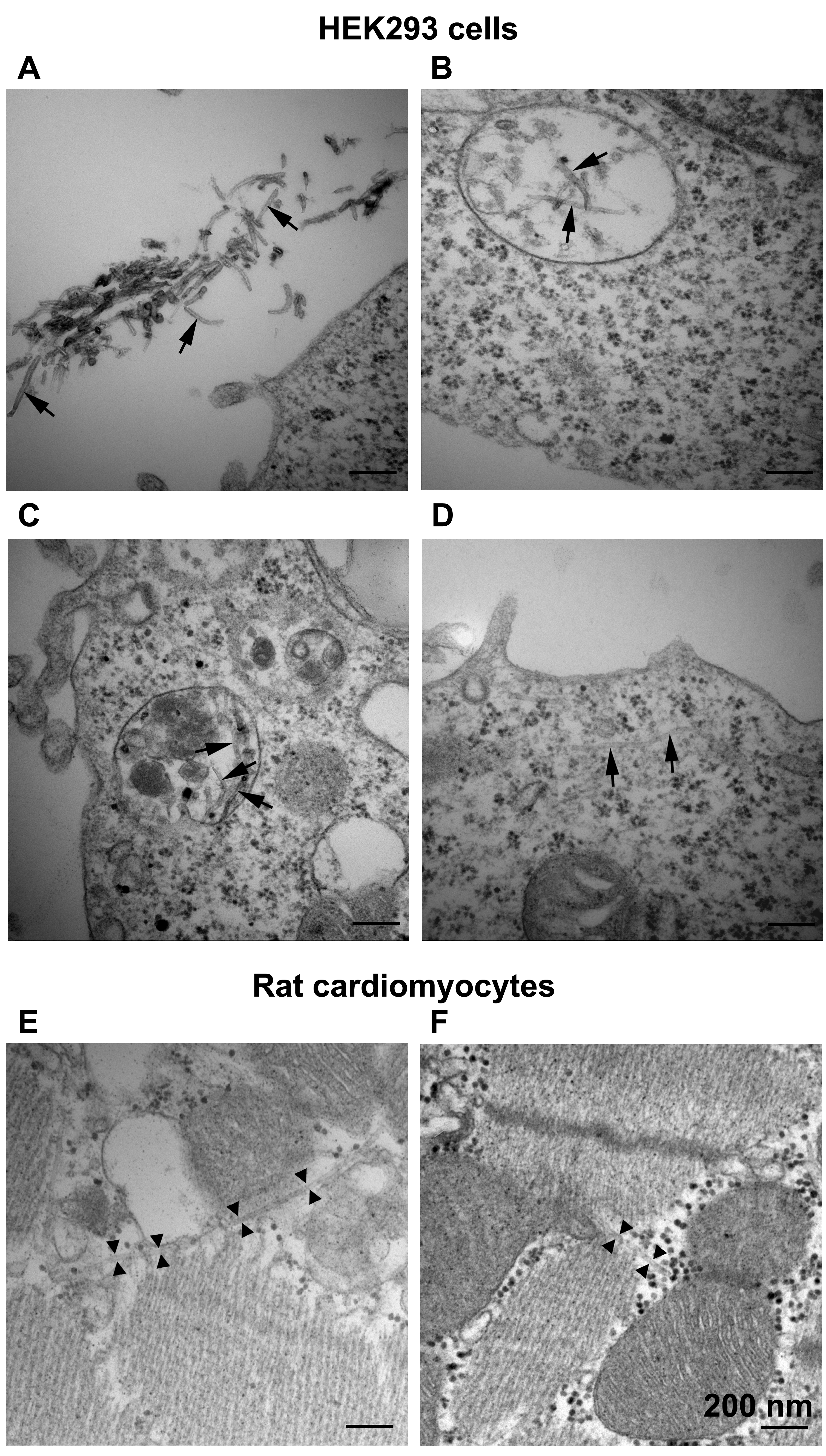

Supplement: Figure S1 — Transmission electron microscopy (TEM) images showing the internalization of MWCNTs in HEK293 cells and cardiomyocytes. A, extracellular MWCNTs showing the appearance of this type of CNT under the transmission electron microscope (arrows). B and C, MWCNTs presented inside the endosomes of HEK293 cells (arrows). D, MWCNTs presented in the cytoplasm of HEK293 cells (arrows). E and F, MWCNTs located in the cytoplasm of rat LV cardiomyocytes (arrow heads). Scale bars, 200 nm. (TIF) [file pone.0101545.s001.tif]

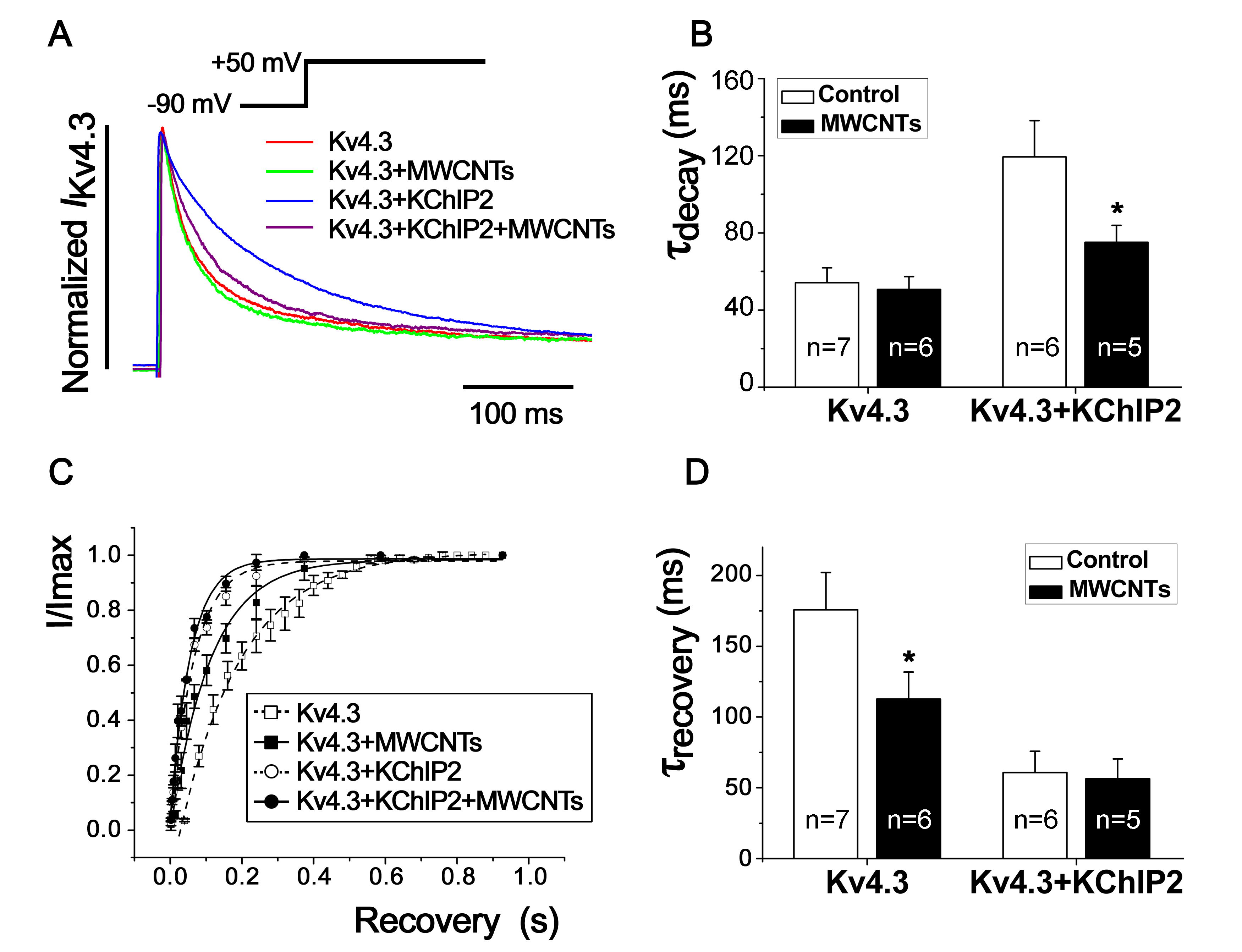

Supplement: Figure S2 — Effects of MWCNTs on the electrophysiological properties of Kv4.3 channel. A, an overlap of normalized I Kv4.3 showing the effects of MWCNTs on the decay kinetics in HEK293 cells expressing Kv4.3 alone or with KChIP2. B, statistical data of decay time constants (τdecay) based on Figure S2A. C, recovery curve fitted by exponential function showing the effects of MWCNTs on the recovery kinetics in HEK293 cells expressing Kv4.3 alone or with KChIP2. D, the statistical recovery time constant (τrecovery) based on Figure S2C. * P<0.05 vs. control. (TIF) [file pone.0101545.s002.tif]

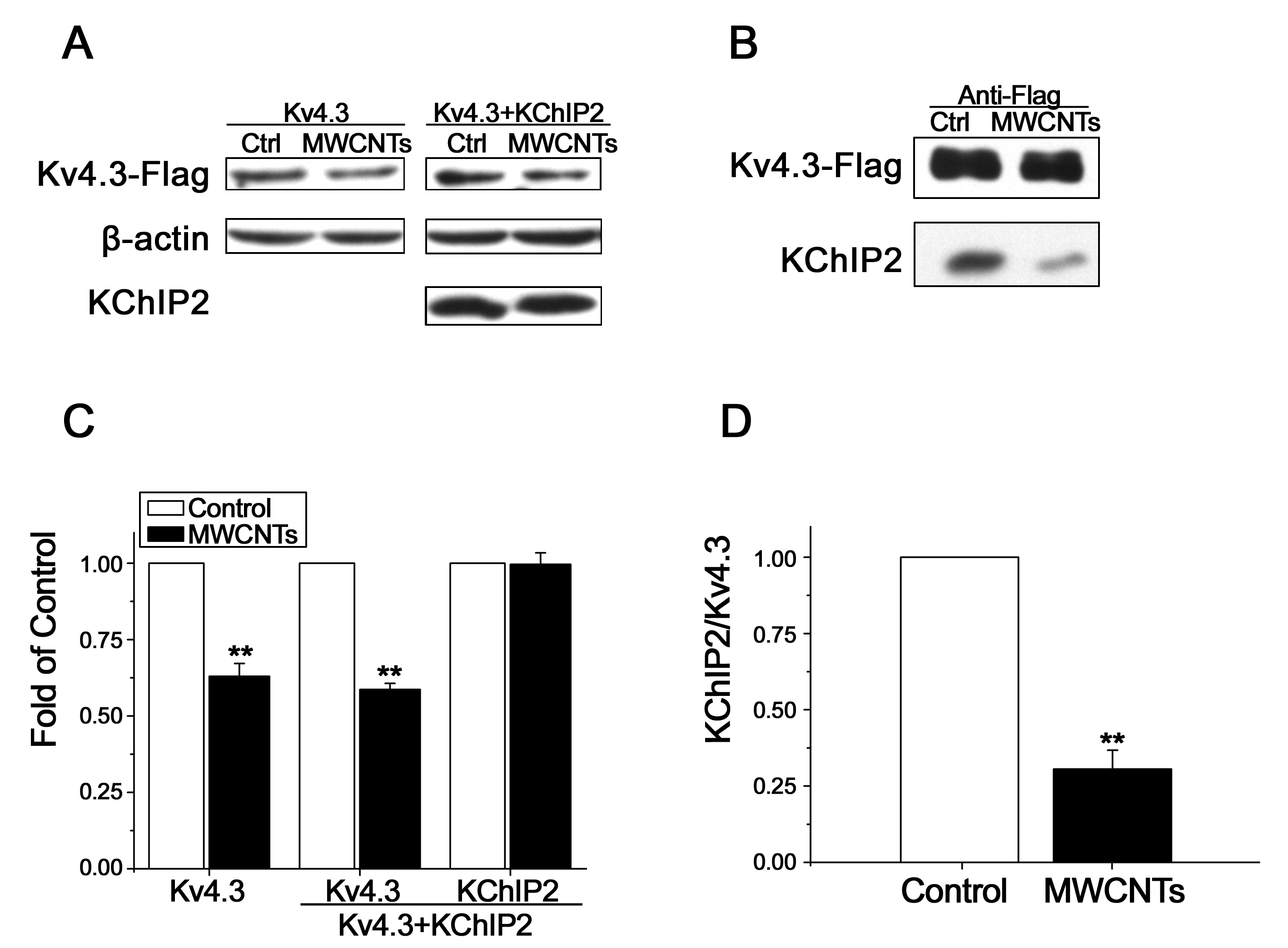

Supplement: Figure S3 — Effects of MWCNTs on the expression of Kv4.3 and on the interaction between Kv4.3 and KChIP2. A and C, Western blotting analysis showing changes of Kv4.3 and KChIP2 in two cell lines and statistical histograms from Figure S3A showing the fold changes after 6 h-treatment with MWCNTs. B, co-IP assay showing the effect of MWCNTs on the interaction between Kv4.3 and KChIP2. The immunoprecipitating antibody (anti-Flag for Kv4.3) was indicated above the lane, and detecting antibodies were shown at the left side. D, statistical results of the effects of MWCNTs on the ratio of KChIP2 to Kv4.3 in HEK293 cells expressing Kv4.3 and KChIP2. ** P<0.01 vs. control. n = 4 for each experiment. (TIF) [file pone.0101545.s003.tif]

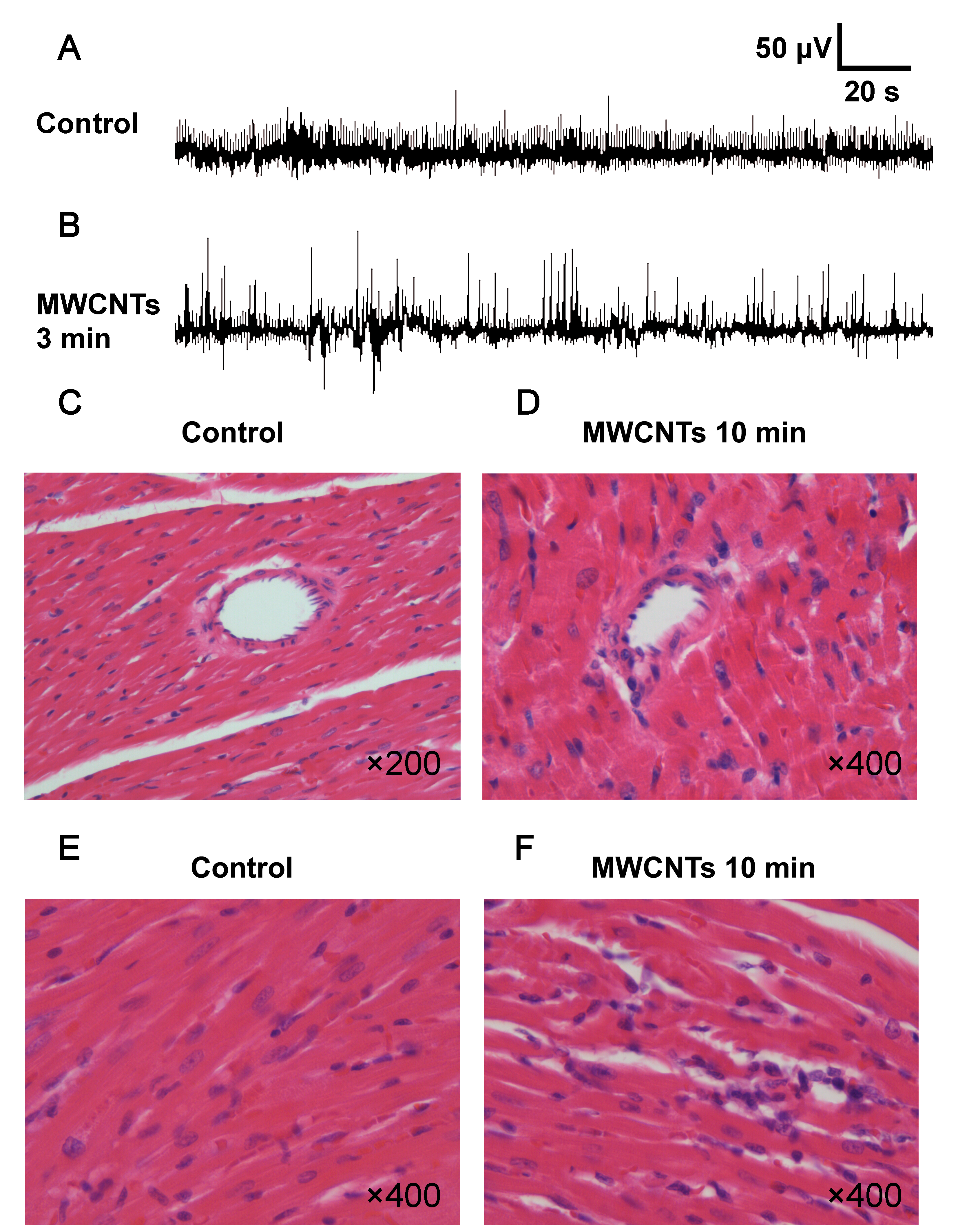

Supplement: Figure S4 — Recording of right cervical vagus discharges and the H&E staining images of rat LV tissues before and after MWCNTs administration in vivo. A and B, vagal discharges before and 3 min after MWCNTs administration in rats in vivo, respectively. Note that vagal discharge was increased by MWCNTs. C through F, hematoxylin and eosin (H&E) staining of rat LV myocardium showing that MWCNTs did not induce coronary occlusion but induced focal myocardial inflammation 10 min after MWCNTs administration. (TIF) [file pone.0101545.s004.tif]
