# Supplementary material for: Multi-Walled Carbon Nanotubes Impair Kv4.2/4.3 Channel Activities, Delay Membrane Repolarization and Induce Bradyarrhythmias in the Rat
Source: PLoS One. 2014 Jul 3;9(7):e101545. doi: 10.1371/journal.pone.0101545 (PMC4081717; doi:10.1371/journal.pone.0101545)
Supplement: Text S1 — Supplementary materials and methods (see in a separate file). (DOC) [file pone.0101545.s005.doc]

**Text S1.** Supplementary materials and methods

*Preparation of surface modified MWCNTs*

We tested three types of MWCNT in this study: the carboxylated MWCNTs (MWCNT-C), aminated MWCNTs (MWCNT-A) and the pristine (electrochemically neutral) MWCNTs (MWCNT-P). The pristine MWCNTs were purchased from Chengdu Organic Chemicals Co. Ltd (Chengdu, China) and the surface modified MWCNTs were prepared as previously described [18]. In brief, the carboxylated MWCNTs (MWCNT-C) were synthesized through a combined oxidation procedure and probe sonication. The as-received MWCNTs were dried at 50°C in a vacuum oven overnight and then were suspended in a 3:1 (volume) mixture of concentrated H_2_SO_4_/HNO_3_ and sonicated with a power of 750 W for 80 sec, the resulting mixture was diluted with a large amount of distilled water, filtered and rinsed thoroughly until pH neutral before drying to constant weight at 50°C in a vacuum oven.

The aminated MWCNTs (MWCNT-A) were obtained through amidation of MWCNT-C with 1,6-diaminohexane. Briefly, 1 mg/ml of MWCNT-C dispersed in 10 ml pure water by the aid of probe sonication was reacted with 10 mg of 1-ethyl-3-(3-dimetylaminopropyl) carbodiimide hydrochloride (EDC, Sigma, USA) and 13 mg N-hydroxysulfosuccinimide sodium salt (Sigma, USA) for 15 min. Then, 6.7 mg of 1,6-diaminohexane dissolved in 1 ml of tetrahydrofuran was added drop by drop and stirred for 2 h at room temperature to have the carboxylic groups amidated with amine groups. The acquired products were purified by dialysis, filtered and washed with pure water, dried in a vacuum oven at 50°C until constant weight.

*Characterization of the modified MWCNTs*

The prepared MWCNT-P, MWCNT-C and MWCNT-A were subjected to scanning electron microscopy (SEM, Hitachi S-5200) to characterize their morphology. The length distribution for the MWCNTs was obtained by counting more than 300 nanotubes randomly taken in ten SEM images. The X-ray photon spectroscopy (XPS, Japan JEOL Scientific JPS-9010TR) and Fourier transform infrared spectroscopy (FTIR, Necolet NEXUS 670) were applied to analyze their surface chemistry. The physicochemical characteristics of surface modified MWCNTs were the same as ones described previously [18]. The prepared MWCNTs showed an outer diameter of 20−30 nm. The average length of pristine MWCNTs was 50 μm. The length distribution for MWCNT-C and MWCNT-A similarly ranges from 300 nm to 1.5 μm, and the average length is 926 nm and 945 nm respectively. XPS analysis indicated that the percentage of carboxylic carbon atoms for carboxylated MWCNTs and amidated carbon atoms for aminated MWCNTs were 4.66% and 2.19%, respectively. FTIR spectrum of MWCNT-C showed that the characteristic absorption of carboxylic group at 1720 cm^-1^ substantially decreased in the MWCNT-A spectrum, while an absorption peak at 1630 cm^-1^ appeared.

*MWCNTs suspension in the culture medium*

The powder sample of the three MWCNTs were sterilized by autoclaving, then dispersed in Dulbecco′s modified Eagle’s medium (DMEM) to obtain a stock solution of 1 mg/ml by the aid of probe sonication (sonication time: 60 seconds, working mode: working 3 seconds following 3 seconds stop; working power was 600 W). For cell study, the MWCNTs were diluted in DMEM at a concentration of 20 μg/ml. For intravenous infusion, 1 mg/ml of MWCNTs were introduced to rats directly.

*Transmission electron microscopy*

Transmission electron microscopy (TEM) was performed to determine the internalization of MWCNTs in HEK293 cells and cardiomyocytes. HEK293 cells and isolated LV myocytes were incubated with MWCNTs for 6 h and then washed with PBS. Cells were then fixed with 2.5% glutaraldehyde in phosphoric buffer and then post-fixed with 1% osmate, dehydrated with gradient ethanol and acetone. Cells were infiltrated by a solution of epoxy resin and acetone (2:1, v/v) overnight, and then by epoxy resin alone for 3 h. Cells were finally embedded in epoxy resin and polymerized at 60°C for 48 h. Ultra-thin sections were cut and mounted on copper grids, stained with uranyl acetate and lead citrate in dark at room temperature, followed by washing with distilled water thoroughly. Ultrastructural images were taken under a transmission electron microscope (JEOL-1011, Japan) at 80 kV with a GATAN digital camera (Gatan, USA).

*Hematoxylin and eosin (H&E) staining*

Hearts were harvested from rats and formalin fixed. Paraffin blocks of myocardium were routinely prepared. Four μm sections were cut with a microtome. Sections were deparaffinized in xylene, rehydrated in graded ethanol and washed with distilled water. Sections were first stained with hematoxylin, washed with water, and then stained with eosin Y. After this staining procedure, sections were washed, dehydrated, cleared and mounted with mounting medium. Images were shot with a DCF digital camera equipped in a light microscope (Leica DM3000, Germany).

*Recording of vagus discharge*

The right-side cervical vagus was isolated and immersed in liquid paraffin to prevent dry. The vagus was cut and a bipolar platinum electrode was placed at the central part of the nerve to monitor vagal output. The discharge signals were recorded with the BL420E data acquisition system (Chengdu Taimeng software Co., Ltd., China). This part of study was to observe the potential effect of MWCNTs on vagal tone.
